# Prediction of high infiltration levels in pituitary adenoma using MRI-based radiomics and machine learning

**DOI:** 10.1186/s41016-022-00290-4

**Published:** 2022-08-12

**Authors:** Chao Zhang, Xueyuan Heng, Wenpeng Neng, Haixin Chen, Aigang Sun, Jinxing Li, Mingguang Wang

**Affiliations:** 1 Department of Neurosurgery, Linyi People’s Hospital, 27 Jiefang Road, Linyi, Shandong 27600 People’s Republic of China; 2 Ebond (Beijing) Intelligence Technology Co., Ltd, Beijing, 100192 People’s Republic of China

**Keywords:** Pituitary adenoma, Machine learning, Preoperative prediction, Magnetic resonance imaging, Infiltration

## Abstract

**Background:**

Infiltration is important for the surgical planning and prognosis of pituitary adenomas. Differences in preoperative diagnosis have been noted. The aim of this article is to assess the accuracy of machine learning analysis of texture-derived parameters of pituitary adenoma obtained from preoperative MRI for the prediction of high infiltration.

**Methods:**

A total of 196 pituitary adenoma patients (training set: *n* = 176; validation set: *n* = 20) were enrolled in this retrospective study. In total, 4120 quantitative imaging features were extracted from CE-T1 MR images. To select the most informative features, the least absolute shrinkage and selection operator (LASSO) and variance threshold method were performed. The linear support vector machine (SVM) was used to fit the predictive model based on infiltration features. Furthermore, the receiver operating characteristic curve (ROC) was generated, and the diagnostic performance of the model was evaluated by calculating the area under the curve (AUC), accuracy, precision, recall, and F1 value.

**Results:**

A variance threshold of 0.85 was used to exclude 16 features with small differences using the LASSO algorithm, and 19 optimal features were finally selected. The SVM models for predicting high infiltration yielded an AUC of 0.86 (sensitivity: 0.81, specificity 0.79) in the training set and 0.73 (sensitivity: 0.87, specificity: 0.80) in the validation set. The four evaluation indicators of the predictive model achieved good diagnostic capabilities in the training set (accuracy: 0.80, precision: 0.82, recall: 0.81, F1 score: 0.81) and independent verification set (accuracy: 0.85, precision: 0.93, recall: 0.87, F1 score: 0.90).

**Conclusions:**

The radiomics model developed in this study demonstrates efficacy for the prediction of pituitary adenoma infiltration. This model could potentially aid neurosurgeons in the preoperative prediction of infiltration in PAs and contribute to the selection of ideal surgical strategies.

**Supplementary Information:**

The online version contains supplementary material available at 10.1186/s41016-022-00290-4.

## Background

Pituitary adenomas are common central nervous system tumors, accounting for approximately 15–20% of all intracranial tumors, with an incidence of 80–90/100,000 [1, 2]. Invasive pituitary adenomas account for approximately 15% of all types of pituitary adenomas. Because these tumors often invade the cavernous sinus, sphenoid sinus, internal carotid artery and suprasellar space, it is more difficult to surgically remove invasive pituitary adenomas compared with noninvasive pituitary adenomas. Moreover, the total resection rate is low, and these tumors easily relapse after surgery and have a poor prognosis [3, 4]. When pituitary adenomas are classified as Knosp grade 3 or 4 on coronal MRI, it has become a common practice to mark them as highly aggressive, and reports have clearly noted that a high Knosp grade (> grade 3) and high recurrence risk are related [5, 6]. In addition, an increasing number of studies use a high Ki67 proliferation index (>3%) as evidence of invasion and proliferation of invasive pituitary adenomas and to distinguish invasive pituitary adenomas from noninvasive pituitary adenomas [7, 8]. However, the Ki67 proliferation index is obtained by immunohistochemical analysis of postoperative pathology.

MRI is the best method for imaging diagnosis of pituitary adenomas. Given the fact that functional pituitary adenomas and nonfunctional pituitary adenomas are initially captured by MRI, the treatment plan is different [9–11]. Therefore, when the brain MRI examination reveals a pituitary adenoma, especially when the tumor is small, if noninvasive MRI can be exclusively used to identify the function of the pituitary adenoma before the operation, this methodology can further identify the high invasiveness and height of the pituitary adenoma before the operation. However, the human eye diagnosis of diagnostic imaging physicians is limited, and it is impossible to identify more subtle tumor features with the naked eye. The emergence of radiomics can precisely help solve this problem.

Radiomics is an emerging field designed to extract a large number of high-dimensional scalable features from medical imaging data (possibly combined with clinical or genomic data) and establish relevant statistical models to assist in diagnostic, prognostic, and therapeutic monitoring. The radiomics workflow includes imaging, ROI segmentation, feature extraction and analysis. Then, statistical models are subsequently designed based on machine learning algorithms that must be adjusted according to clinical or biological issues and the prior knowledge available [12].

This report proposes a method of using radiomics to classify MRI images of pituitary tumors based on infiltration. We aim to use machine learning algorithms to develop a radiomics model that predicts the high invasiveness of pituitary adenomas, providing a new preoperative evaluation method for patients with pituitary adenomas to better formulate treatment strategies.

## Methods

### Patients

Ethical approval for this retrospective analysis was obtained from the science research ethics committee of Linyi People’s Hospital, and the need for informed consent was waived. All patients with pituitary tumors who underwent surgical resection at our hospital from February 2016 to July 2020 were enrolled.

**Fig. 1:**
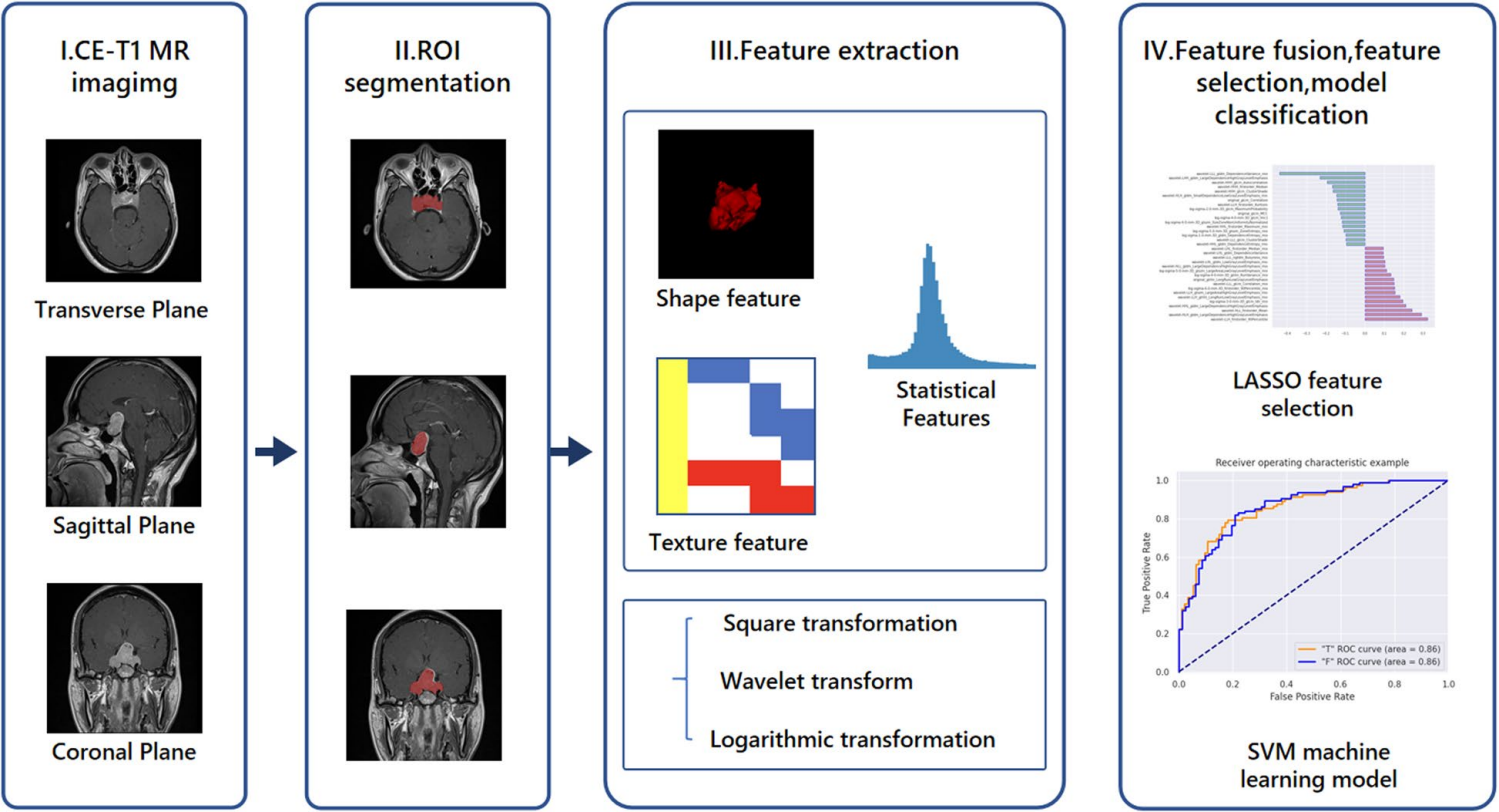
Flow chart of this study. **I** Three scanning positions of the original CE-T1 image: axial, sagittal, and coronal. **II** Segmentation of ROI. **III** Transform after extracting features from ROI. **IV** Feature selection and model establishment

We collected clinical information of patients, including age, sex, preoperative computer MRI images, bilateral Knosp classification, preoperative hormones, endocrine symptoms, postoperative pathology, and immunohistochemical results. According to the relationship between cavernous sinus and the adenoma during operation, we define invasiveness tumors as highly aggressive and others as not highly aggressive. A Ki67 proliferation index > 3% was defined as a high Ki67 proliferation index [5, 7]. The following inclusion criteria were employed: (1) the quality of preoperative computer MRI images is good, images lack artifacts, and the images are from the same MRI scanner; (2) complete preoperative hormone examination; and (3) all patients were pathologically confirmed as having pituitary adenomas based on immunohistochemistry. The following exclusion criteria were applied: (1) patients who received previous surgery, drugs, or radiotherapy, and (2) preoperative computer MRI images have obvious artifacts. A dataset containing 196 patients was obtained (93 males and 103 females, average age: 52 ± 12.98 years, age range 11–76 years). The dataset is divided into three scan bits, including coronal plane, transverse plane, and sagittal plane, with 198 samples of coronal plane and sagittal plane scans and 196 transverse plane samples. The training set (*n*=176) and the validation set (*n*=20) are divided in a ratio of 9:1. Table 1 lists the specific characteristics of the patients in the training set and validation set. The training set is used for image feature construction and model development, and the validation set is used for model verification. Our flow chart is shown in Fig. 1.

**Table 1:** Patients clinical characteristics (*n*=196)

Characteristic	Training set (*n*=176)	Validation set (*n*=20)	Whole set (*n*=196)	*P-value*
Age (year, mean ± std)	52.43 ± 12.82	48.70 ± 14.05	51.99 ± 12.98	0.224
Gender				0.809
Male	83 (47.16%)	10 (50.00%)	93 (47.45%)	
Female	93 (52.84%)	10 (50.00%)	103 (52.55%)	
Knosp grade				0.608
Grades 0–2	81 (46.02%)	8 (40.00%)	89 (45.41%)	
Grades 3–4	95 (53.98%)	12 (60.00%)	107 (54.59%)	
Hormone hypersecreting tumors				0.707
Yes	87 (49.43%)	9 (45.00%)	96 (48.98%)	
No	89 (50.57%)	11 (55.00%)	100 (51.02%)	
Infiltration				0.419
High invasion	95 (53.98%)	12 (60%)	107 (60.79%)	
Not high invasion	81 (46.02%)	8 (40%)	99 (39.21%)	
Ki67 proliferation index				0.103
< 3%	120 (68.18%)	10 (50.00%)	130 (66.33%)	
≥ 3%	56 (31.82%)	10 (50.005)	66 (33.67%)	

*std* standard deviation. *P* value < 0.5 represents a significant difference

### MRI image acquisition

All patients underwent saddle area scans using the same MRI scanner (Siemens, 3.0T, Trio, Germany) in our hospital. The imaging schemes included T1-weighted imaging (T1WI), T2-weighted imaging (T2WI), and CE-T1. Given that the image resolution of each slice that CE-T1 may obtain is very high, the contrast difference between normal tissue and tumor tissue will increase after the injection of the contrast agent [13]. Therefore, in this study, we selected the patient images from the archive system in our hospital. The CE-T1 image is used for analysis. The collection and setting parameters of the CE-T1 sequence are as follows: repetition time/echo time (TR/TE) = 232/8.1 ms, slice thickness=2 mm, field of view (FOV) = 200 × 200 mm^2^, and voxel size = 0.9 × 0.6 × 2.0 mm^3^.

### Tumor segmentation

After the MRI image was examined by a radiologist, the ROI of all tumors was segmented by two neurosurgeons and radiologists with more than 10 years of experience in pituitary adenoma diagnosis and treatment. Two radiologists (experts 1 and 2 with 11 and 27 years of experience in brain MRI, respectively) delineated ROIs while blinded to the pathology results using 3D Slicer (https://www.slicer.org). Intersection over union (IOU) was calculated to determine the agreement between two radiologists, thus evaluating interobserver reproducibility. For cases with an IOU greater than 0.8, we chose the delineation result from expert 1 as a reference, and the ROIs of the remaining cases were then determined by a third experienced radiologist. Under double-blind conditions, 3D Slicer software was used to analyze the coronal position (COR), and sagittal (SAG) and axial (TRA) CE-T1 images were manually depicted. Disagreements arising during the analysis were resolved by the two physicians through negotiation. Finally, 588 ROIs were divided from 196 coronal, sagittal, and axial samples for subsequent radiomic analysis. In addition, the two physicians evaluated the Knosp classification of all samples in the coronal image under double-blind conditions, and the results were consistent.

### Feature dimension reduction

**Fig. 2:**
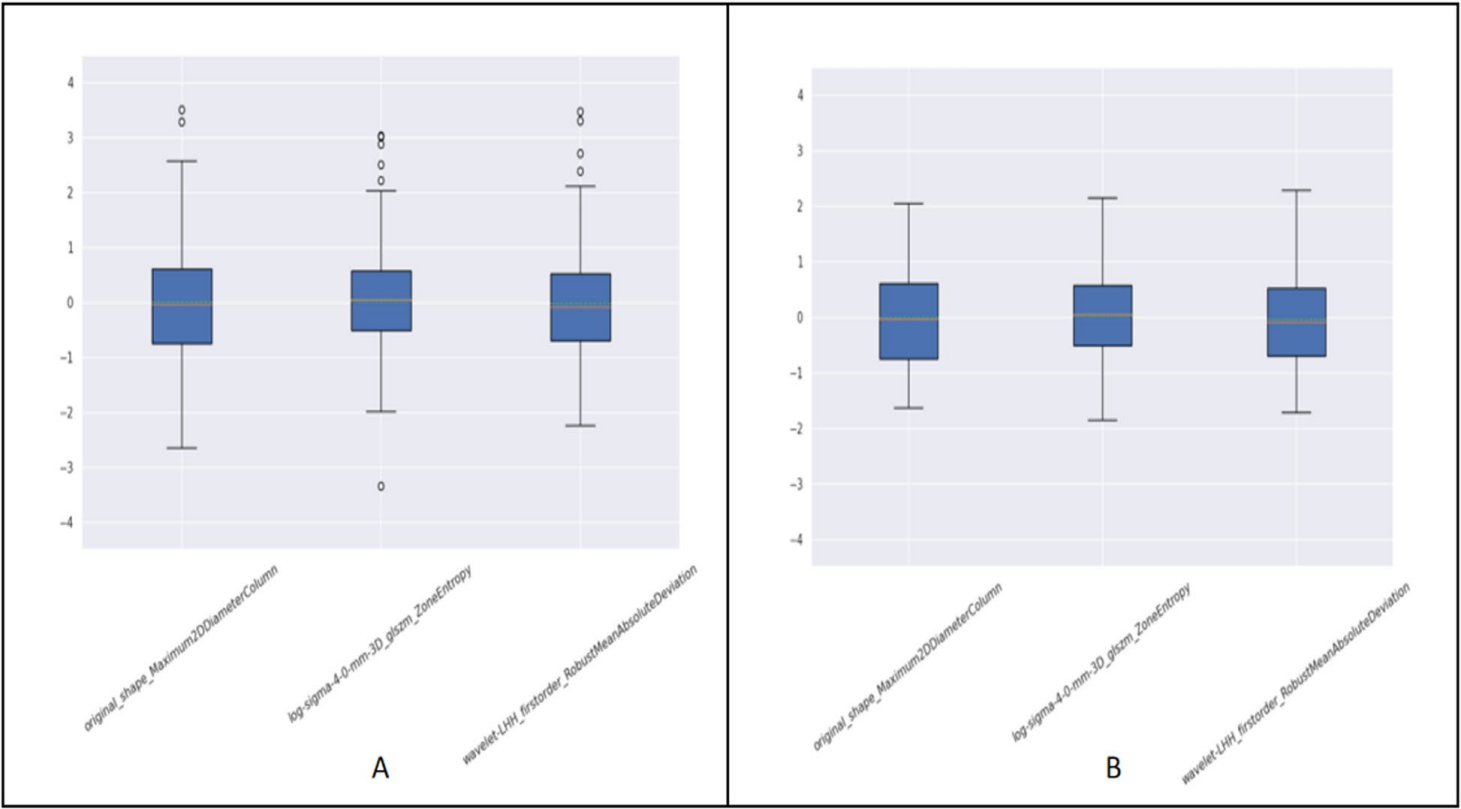
The contrast of handles outlier points. **A** The distribution of does not process outlier point. **B** The distribution of processes outlier point

**Fig. 3:**
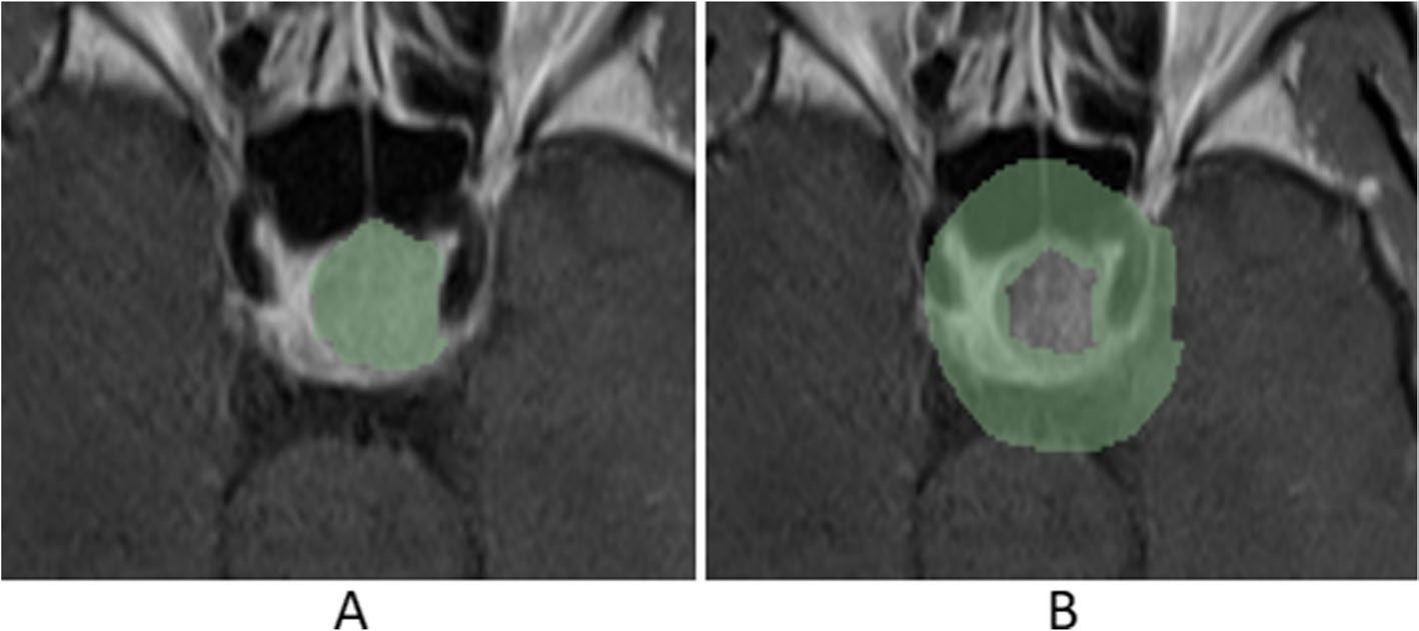
Mask of the area surrounding the tumor. **A** Mask ROI. **B** mask_edge ROI

1. Image value processing: The voxels of the MRI image exhibit large variations over the numerical range. Therefore, it is particularly important to reduce the variation of the dataset MRI images. The image value processing method used in this classification task is as follows: Assuming that the set of all voxel values of an image is *X*, the 99% quantile *P* of pixel values in the image region is taken, and the image scaling value *M* is defined. The pixel values in the image are calculated based on the following equation: window area = (*X*·*M*)/*P*. In addition, the Xwin voxel value greater than *M* is equal to *M*.
2. Processing outlier points: When counting a feature of all samples, some feature points deviate far from the overall distribution, which affects the classification effect of subsequent models, so these outlier points are treated as follows. Assuming that a set of a feature of the whole sample is *F*, to take the 99.9% quantile P 99.9, the *F* is set to a value greater than P 99.9 values, and values are all equal to P 99.9. The eigenvalue point distribution of the nonprocessed data (Fig. 2A) versus the processed data (Fig. 2B) is described as follows:
3. Resampling: To unify the spacing of all images to [0.625, 0.625, 2.4].
4. Multiple ROIs: For this classification task, certain pathologies associated with infiltration were present in the surrounding areas of the tumor; therefore, the task adopted a mask in the original tumor region. The mask from the area surrounding the tumor was also used to obtain this mask. Using the SimpleITK kit or Opencv kit, corrosion operations were performed on the original mask. Mask_e was obtained to expand the original mask, and mask_d was obtained. By subtracting mask_e from mask_d, the mask of the area surrounding the tumor was obtained (Fig. 3).

### Preprocessing used for classification task

We used a preprocessing method for the infiltration classification task (Additional file 1).

### Feature extraction

Quantitative image features were extracted from MRI images using the Pyradiomics library (wavelet parameters: rbio1.1, LoG parameters for image value processing: original-mask sigma = 1.0, 2.0, 3.0, 4.0, 5.0, and 6.0; edge-mask sigma = 1.0, 2.0, 3.0, 4.0, 5.0, and 6.0; LoG parameters for no image value processing: original-mask sigma=2.0, 3.0, 4.0, 5.0; edge-mask sigma=1.0, 2.0, 3.0, 4.0, 5.0, and 6.0) in Additional file 2.

### Feature dimension reduction

As described above, a large number of image features can be obtained. However, most of these features may be of no use for classification tasks. Therefore, it is necessary to reduce the features and identify features with high pathological correlation. To reduce redundancy, feature reduction was performed using the following methods:

The variance threshold method removes the eigenvalues with a variance less than the threshold. Using the LASSO model, L1 is regularized as a cost function with alphas=0.001 and a maximum of 10000 iterations.

### Statistical analysis (classifier)

After feature screening, there are multiple supervised learning classifiers available for classification analysis based on the selected features. In this study, a model based on Radiomics is constructed using a linear support vector machine (LinearSVM) classifier, and the effectiveness of the model is verified. Parameter settings for the linear support vector machine LinearSVM are noted as follows: infiltration: C = 1, random_state = 23, and max_iter = 10000. To evaluate predictive performance, the operating feature (ROC) curve and curve area (AUC) were used in the training and validation datasets. The study uses five indicators to evaluate the performance of the classifiers: (1) accuracy (ACC), (2) precision (P) ((precision = true positives/(true positives + false-positives)), (3) Recall ® (recall = true positives/(true positives + false negatives)), (4) F1 Score (f1 score = P*R*2/(P + R)), and (5) support (the total number of samples in the test set).

## Results

### Clinical characteristics

A total of 196 patients (age, 51.99 ± 12.98 years) were enrolled in this study. The characteristics of the patients and tumors are shown in Table 1. No significant differences for all clinic-radiological factors (*p* = 0.103-0.809) were found between the training set and validation set, thereby justifying the use of the training set and validation set.

### Statistical results of the datasets

The label distribution of the validation sets and training sets for different classification tasks is listed in Table 1. In total, 10% of all cases (male to female ratio: 10:10) were included in the validation set, and 90% of all cases (male to female ratio: 83:93) were included in the training set. In the validation set, 12 cases were highly invasive, accounting for 60% of all cases. Eight cases were not highly invasive, accounting for 40% of all cases. In the training test, 95 cases were highly invasive, accounting for 54% of all cases, and 81 cases were not highly invasive, accounting for 46% of all cases.

### Feature screening results

**Fig. 4:**
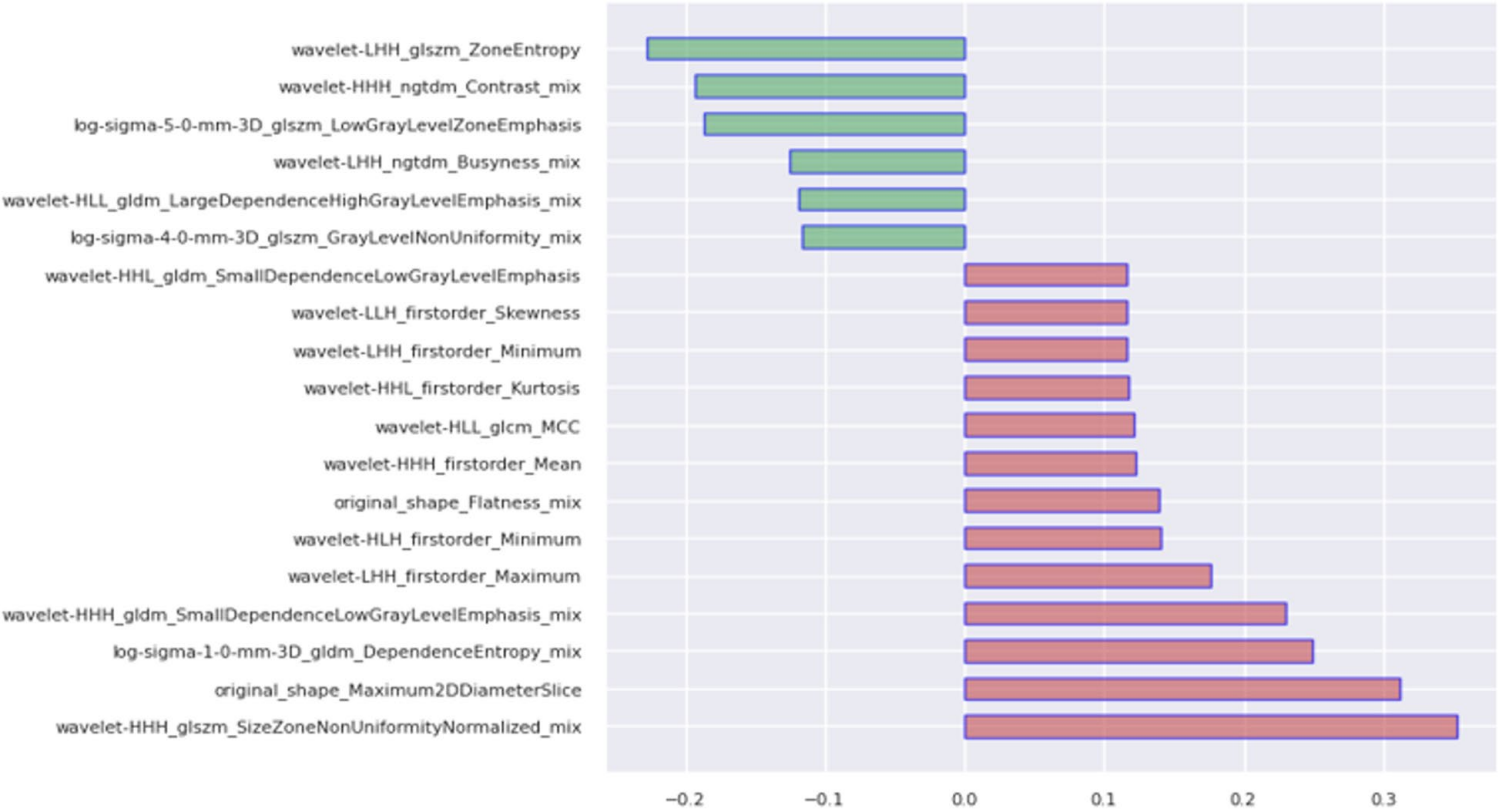
Weights obtained by lasso method in the classification task with high infiltration

A total of 4120 quantitative imaging features were extracted from the three scanning positions of contrast-enhanced T1-weighted imaging (CE-T1). For highly invasive classification tasks, a variance threshold of 0.85 was used to exclude 16 features with small differences using the LASSO algorithm (Fig. 4), and 19 optimal features were finally selected (Table 2). The following lasso parameter settings were employed: alphas=0.001, max_iter (10000).

**Table 2:** Features selected for validation classification task

Radiomic feature	Radiomic class	Filter	Mask
Maximum2DDiameterSlice	shape	original	original
LowGrayLevelZoneEmphasis	glszm	log-sigma-5-0	original
Skewness	firstorder	wavelet-LLH	original
Maximum	firstorder	wavelet-LHH	original
Minimum	firstorder	wavelet-LHH	original
ZoneEntropy	glszm	wavelet-LHH	original
MCC	glcm	wavelet-HLL	original
Minimum	firstorder	wavelet-HLH	original
Kurtosis	firstorder	wavelet-HHL	original
SmallDependenceLowGrayLevelEmphasis	gldm	wavelet-HHL	original
Mean	firstorder	wavelet-HHH	original
Flatness	shape	original	egde
DependenceEntropy	gldm	log-sigma-1-0	egde
GrayLevelNonUniformity	glszm	log-sigma-4-0	egde
Busyness	ngtdm	wavelet-LHH	egde
LargeDependenceHighGrayLevelEmphasis	gldm	wavelet-HLL	egde
SizeZoneNonUniformityNormalized	glszm	wavelet-HHH	egde
Contrast	ngtdm	wavelet-HHH	egde
SmallDependenceLowGrayLevelEmphasis	gldm	wavelet-HHH	egde

Features selected for validation classification task

### Model performance

**Fig. 5:**
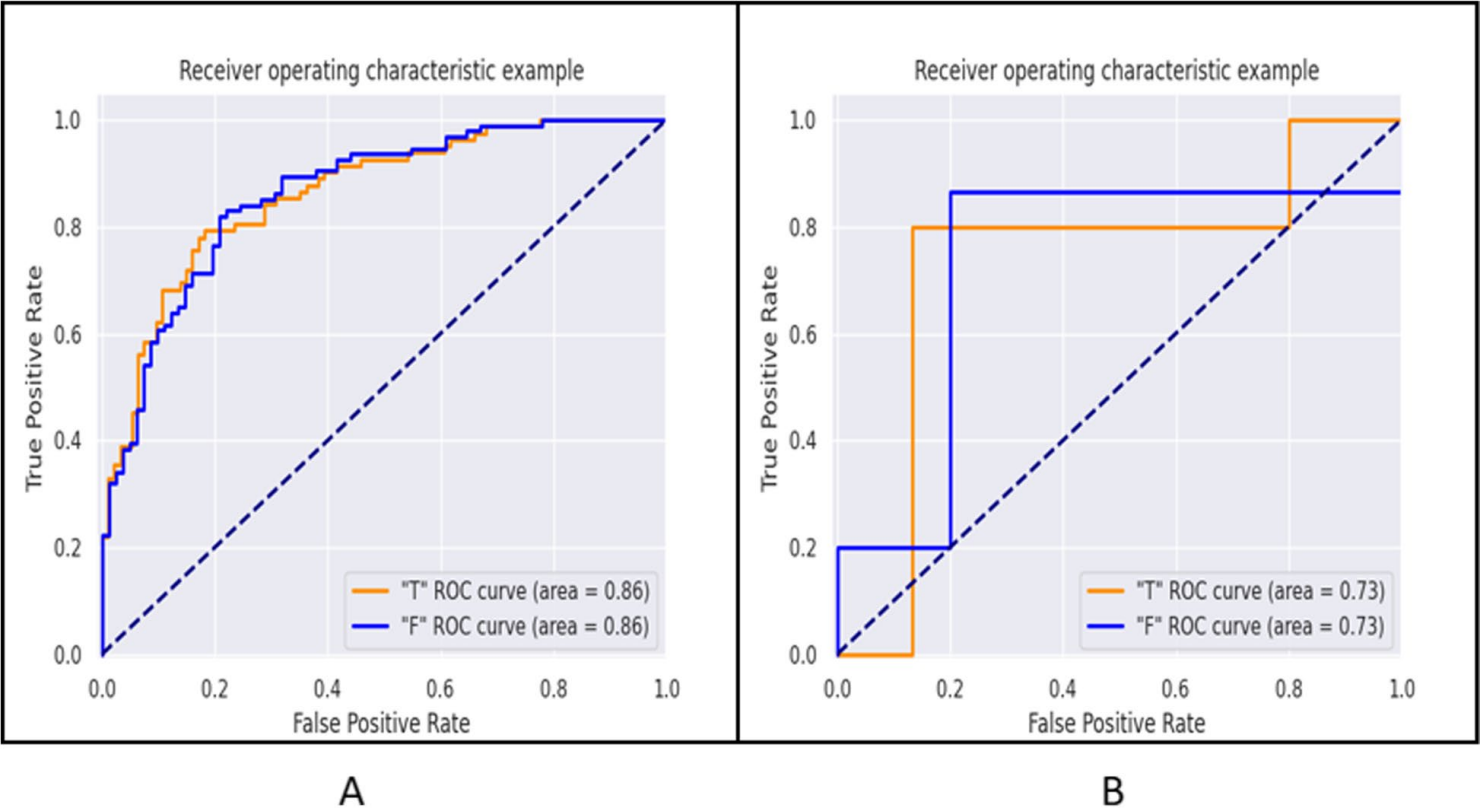
ROC curve of high infiltration classification task. **A** Training set. **B** Validation set (“T” stands for “Yes,” “F” stands for “None”)

Figure 5 shows the ROC curves of the classification task. Table 3 shows the AUC, sensitivity, and specificity of different classification tasks in the training set and validation set (“T” represents “Yes,” “F” represents “None”). The radiomics model showed good discrimination. For highly invasive classification tasks, the AUC of the training set was 0.86 (95% confidence interval: 0.75–1.00), the sensitivity was 0.81, and the specificity was 0.79. The AUC of the validation set was 0.73 (95% confidence interval: 0.53–0.94), the sensitivity was 0.87, and the specificity was 0.80. The results show that the radiomics model we established is feasible for distinguishing highly aggressive pituitary adenomas.

**Table 3:** Results of ROC curve analysis and five indicators in the training set and verification set

	Training set		Verification set	
	Indicators	Infiltration	Indicators	Infiltration
**T**	**AUC**	0.86	**AUC**	0.73
	**95% CI**	0.75–1.00	**95% CI**	0.53–0.94
	**Sensitivity**	0.81	**Sensitivity**	0.87
	**Specificity**	0.79	**Specificity**	0.80
	**Accuracy**	0.8	**Accuracy**	0.85
	**Precision**	0.82	**Precision**	0.93
	**Recall**	0.81	**Recall**	0.87
	**F1-score**	0.81	**F1-score**	0.90
	**Support**	94	**Support**	15
**F**	**AUC**	0.86	**AUC**	0.73
	**95% CI**	0.75–1.00	**95% CI**	0.53–0.94
	**Sensitivity**	0.79	**Sensitivity**	0.80
	**Specificity**	0.81	**Specificity**	0.87
	**Accuracy**	0.8	**Accuracy**	0.85
	**Precision**	0.78	**Precision**	0.67
	**Recall**	0.79	**Recall**	0.80
	**F1-score**	0.79	**F1-score**	0.73
	**Support**	82	**Support**	5

### The classifier evaluation results

We summarized the four evaluation indicators of the prediction model (accuracy rate, precision rate, recall rate, and F1 value). As shown in Table 3, the model achieved good diagnostic capabilities in the training set and independent verification set. Specifically, the accuracy was 0.80, the precision was 0.82, the recall was 0.81, and the F1 score was 0.81 in the training set. In addition, the accuracy was 0.85, the precision was 0.93, the recall was 0.87, and the F1 score was 0.90 in the independent verification set.

## Discussion

Based on MRI, this study used radiomics to develop a radiomics model that predicts the high invasiveness of pituitary adenomas and achieved good results. The results demonstrate that the radiomics model predicts the invasiveness of pituitary adenomas. The model exhibits good potential for predicting highly invasive pituitary adenomas. This information may help clinicians assess the nature of the tumor before surgery and better formulate treatment strategies.

The concept of radiomics was first proposed by Dutch scholar Philippe Lambin and has been applied to the prediction and evaluation of a variety of tumors, such as colorectal cancer, lung cancer, and prostate cancer [14–19]. In recent years, radiomics has also been confirmed to exhibit increasing potential in the preoperative diagnosis of pituitary adenomas. The radiomics model established by Zhang et al. is used to distinguish subtypes of nonfunctional pituitary adenomas before surgery, and the model performs well [2]. Fan et al. applied radiomics to predict the response of patients with acromegaly to radiotherapy [20]. Niu et al. developed a radiomics model that predicted the invasion of pituitary adenomas on the cavernous sinus [21]. Peng et al. [22] developed a machine learning model for predicting the immunohistochemical classification of pituitary adenomas before surgery.

In this study, based on preoperative CE-T1 images of patients with pituitary adenomas and using machine learning methods, we developed a radiomics model that predicts the high invasiveness of pituitary adenomas. The good AUC value of the final training set and the validation set demonstrated its potential value in the prediction of high invasiveness of pituitary adenoma, and its reliability was verified by calculating the index of the classifier. Niu et al. identified a radiomics nomogram based on CE-T1 and T2 MR images for the individualized evaluation of cavernous sinus invasion in 194 patients with PAs (Knosp grades two or three) [21]. The radiomics model yielded area under the curve (AUC) values of 0.852 and 0.826 for the training and test sets, respectively. In contrast, our study enrolled patients to predict high invasiveness (cavernous sinus invasion). We calculated the area under the curve (AUC), accuracy, precision, recall, and F1 value to evaluate the diagnostic performance, but the performance of the two models needs to be further compared.

A very important issue in the application of radiomics is the selection of the best features. Unlike some previous studies [15, 16, 18, 23, 24], our research uses some innovative methods. First, in the application of image data, our study takes into account the clinical domain knowledge. For ROI segmentation, three window positions, including coronal, axial, and sagittal, were included to minimize errors and improve three-dimensional features. Second, regarding the microenvironment around the tumor, a mask expansion method is used when preprocessing the data. Third, in the feature extraction method, filter transformation is used in addition to the original features to extract finer texture features. These results laid the foundation for the good performance of the model.

Our research has some limitations. First, our research was conducted at a single center. All image data were obtained from the same machine in a hospital. Whether our model is suitable for multicenter research remains to be demonstrated. In the future, we need to test the imaging data from different centers to validate our model. In addition, all ROI annotations are performed manually, which is time-consuming. Given the availability of more sample sets in the future, if an automatic segmentation algorithm can be created, an end-to-end application can be formed. Therefore, we need to identify an accurate automatic segmentation algorithm. Third, research has revealed that the consistency of T2-weighted images may be better25. In our study, only CE-T1 images were used, and no comparison with other sequences was performed.

## Conclusion

In summary, we used radiomics to create a predictive model of highly invasive pituitary adenomas, and independently verified its reliability in the validation set. In the future, the number of samples will be increased, and the model will be continuously optimized. The diagnostic ability of the model is expected to be further improved, and this model may help neurosurgeons formulate better treatment strategies.

## Supplementary Information

**Additional file 1.**

**Additional file 2.**

## Data Availability

The datasets used and analyzed during the current study are available from the corresponding author on reasonable request.

## Abbreviations

ACC: Accuracy
AIC: Akaike’s information criterion
AUC: Area under the curve
BIC: Bayesian information criterion
CE-T1: Contrast-enhanced T1-weighted
CI: Confidence interval
CS: Cavernous sinus
DCA: Decision curve analysis
FOV: Field of view
LASSO: Least absolute shrinkage and selection operator
MRI: Magnetic resonance imaging
PAs: Pituitary adenomas
ROC: Receiver operating characteristic curve
ROIs: Regions of interests
SSA: Somatostatin analogs
SVM: Support vector machine
T1WI: T1-weighted imaging
TR/TE: Repetition time/echo time
