# Supplementary material for: Prediction of high infiltration levels in pituitary adenoma using MRI-based radiomics and machine learning
Source: Chin Neurosurg J. 2022 Aug 12;8:21. doi: 10.1186/s41016-022-00290-4 (PMC9373412; doi:10.1186/s41016-022-00290-4)
Supplement: Supplementary file 1 — Additional file 1. [file 41016_2022_290_MOESM1_ESM.docx]

Additional file 1

Preprocessing methods used for invasion classification task

| **Label** | **Scan the bit** | **Image value**  **processing** | **Outlier-point processing** | **Resample** | **Edge mask extraction** |
| --- | --- | --- | --- | --- | --- |
| **High Invasion** | **TRA** | **×** | **√** | **√** | SimpleITK (15,15,0) (3,3,0) |
