# Supplementary material for: Prediction of high infiltration levels in pituitary adenoma using MRI-based radiomics and machine learning
Source: Chin Neurosurg J. 2022 Aug 12;8:21. doi: 10.1186/s41016-022-00290-4 (PMC9373412; doi:10.1186/s41016-022-00290-4)
Supplement: Supplementary file 2 — Additional file 2. [file 41016_2022_290_MOESM2_ESM.docx]

Additional file 2

Feature extraction methods for functionality classification task

|  | **Feature** | **Functional** |
| --- | --- | --- |
| **Filter** | Original | √ |
|  | Wavelet | √ |
|  | LoG | √ |
|  | LBP-3D | × |
| **Class** | FirstOrder | √ |
|  | Shape3D | √ |
|  | GLCM | √ |
|  | GLSZM | √ |
|  | GLRLM | √ |
|  | NGTDM | √ |
|  | GLDM | √ |
| **Mask** | Original | √ |
|  | Edge | √ |
